# KT‐939: A Next‐Generation Human Tyrosinase Inhibitor With Superior Efficacy for the Safe Management of Hyperpigmentation

**DOI:** 10.1111/jocd.70510

**Published:** 2025-10-29

**Authors:** Xiaodan Hou, Yunhui Li, Qun Zhang, Honghua Yan, Xin Zhang, Yueying Yang, Xiang Cai, Jin Wang, Miaohua Ding, Dong Chen, Jie Chen, Youzhi Tong, Nina Mehta, Andy Goren

**Affiliations:** ^1^ Kintor Pharmaceutical Limited Suzhou Jiangsu China; ^2^ The University of North Carolina School of Medicine Chapel Hill North Carolina USA; ^3^ University of Rome “G. Marconi” Rome Italy

**Keywords:** hyperpigmentation, KT‐939, melanogenesis, tyrosinase

## Abstract

**Background:**

Tyrosinase is the rate‐limiting enzyme in melanin biosynthesis, and its overactivity contributes to hyperpigmentation disorders. Existing tyrosinase inhibitors are often limited by poor potency against human tyrosinase (hTYR) or safety concerns.

**Aims:**

To evaluate the inhibitory potency, safety, and multifunctional activity of KT‐939, a newly synthesized human tyrosinase inhibitor, compared with established depigmenting agents.

**Patients/Methods:**

KT‐939 was synthesized and tested in vitro for tyrosinase inhibition, melanin suppression in human melanocytes, antioxidant activity (DPPH radical scavenging, NRF2 pathway activation), and anti‐inflammatory activity (cytokine expression in LPS‐stimulated macrophages). Safety was assessed in multiple skin‐related cell lines. A 28‐day, single‐center clinical study in healthy women with sensitive skin assessed the effects of 0.2% KT‐939 lotion on pigmentation and tolerability.

**Results:**

KT‐939 strongly inhibited hTYR (IC₅₀ = 0.07 μM), demonstrating ~4‐fold greater potency than Thiamidol and far surpassing other comparators. In melanocytes, KT‐939 reduced melanin production (IC₅₀ = 0.36 μM) with reversible effects upon withdrawal. KT‐939 also displayed antioxidant activity, NRF2 activation, and suppression of pro‐inflammatory cytokines, without cytotoxicity up to 50 μM. Clinically, 28 days of KT‐939 lotion use improved skin spot lightening, tone uniformity, and overall brightness, with good tolerability in sensitive skin.

**Conclusions:**

KT‐939 is a potent and safe human tyrosinase inhibitor with additional antioxidant and anti‐inflammatory activity. These findings support its potential in cosmetic skin brightening and as a therapeutic candidate for hyperpigmentation disorders.

## 1 Introduction

Melanin synthesis and its deposition within melanocytes not only protects the skin from ultraviolet radiation and prevents tissue overheating, but also plays an important role in maintaining skin health, physiological balance, and visible appearance. When the skin is exposed to external stimuli (e.g., prolonged ultraviolet exposure, infections, allergies, excessive exfoliation, skincare products containing heavy metals, or environmental pollution) or internal factors (e.g., emotional stress, endocrine disorders, reduced metabolic rate, hormonal changes, or genetic predisposition), melanin overproduction may be triggered, leading to pigmentary disorders including freckles, age‐related spots, melasma, sunspots, and inflammation‐induced hyperpigmentation [1, 2]. These conditions significantly affect facial appearance and the quality of life of patients. Extensive medical and experimental studies have confirmed that tyrosinase serves as the key rate‐limiting enzyme in melanin synthesis [3]. Increased tyrosinase activity accelerates melanin formation, resulting in an accumulation of melanin that cannot be efficiently metabolized and cleared, thereby causing hyperpigmentation. Inhibition of tyrosinase activity effectively suppresses excessive melanin synthesis and represents an important strategy for the prevention and treatment of hyperpigmentation‐related conditions [4].

Over the past few decades, numerous natural and synthetic tyrosinase inhibitors have been reported, including arbutin, hydroquinone, kojic acid, and various aromatic compounds such as benzaldehydes, benzoic acids, cycloheptatrienone, and polyphenols [5, 6]. Based on their mode of action, these inhibitors can be categorized as competitive, non‐competitive, or mixed‐type inhibitors [4]. Despite the identification of many inhibitors, only a limited number (e.g., kojic acid, arbutin, cycloheptatrienone, and resveratrol) are currently used in cosmetics or as therapeutic agents [7]. Most tyrosinase inhibitors have been evaluated using mushroom tyrosinase (mTYR) as the model enzyme; however, significant structural and biochemical differences exist between mTYR and human tyrosinase (hTYR) [8]. Specifically, mTYR is a soluble oligomeric enzyme located in the cytoplasm, whereas hTYR is a membrane‐associated, highly glycosylated monomer predominantly localized within melanosomes. The amino acid sequence homology between hTYR and mTYR is only 22%–24%, and their active sites exhibit notable differences [9]. Consequently, many mTYR inhibitors that are effective against mTYR show markedly reduced potency when tested against hTYR. This includes some widely used tyrosinase inhibitors in whitening agents, which show inhibition constants (Ki) or half‐maximal inhibitory concentrations (IC_50_) for hTYR that are 100–1000 times higher, indicating much lower potency compared with mTYR [10]. Resorcinol derivatives, which have a similar structure to tyrosinase substrates, can effectively inhibit tyrosinase activity and have efficient whitening effects. Currently, widely used resorcinol derivatives in the market include isobutylamido thiazolyl resorcinol (Thiamidol), 4‐butylresorcinol (4BR), hexyl resorcinol (HR), phenethyl resorcinol (377), 2,4‐dimethoxybenzyl resorcinol (CL302), and glabridin [10, 11, 12].

Skin whitening ingredients are typically recommended for long‐term use; however, they may cause various side effects such as redness, itching, tingling, and uneven skin tone [13, 14]. The development and combination of antioxidant and anti‐inflammatory agents show considerable potential in skin whitening and freckle reduction, offering consumers broader and safer treatment options [2, 15, 16]. Consequently, identifying effective and well‐tolerated treatments to address pigment irregularities and prevent the recurrence of hyperpigmented spots remains a critical focus in pigmentation research. Based on our previous studies, we chemically synthesized a series of small molecule inhibitors targeting hTYR and successfully identified KT‐939, a candidate with superior potency and safety. In this study, we investigated the effects of KT‐939 on tyrosinase activity and melanin production in human melanocytes in vitro. For comparison, the inhibitory effects of established skin‐lightening agents including arbutin, hydroquinone, kojic acid, phenylethyl resorcinol, 4‐butylresorcinol, and Thiamidol were also evaluated. In addition to effectively inhibiting melanin production, KT‐939 exhibited antioxidant and anti‐inflammatory effects. Furthermore, KT‐939 demonstrated effective and safe inhibitory activity against human hyperpigmentation in clinical studies.

## 2 Materials and Methods

### 2.1 Synthesis of *N*‐(4‐(2,4‐Dihydroxyphenyl) Thiazol‐2‐yl)‐3‐Methyloxetane‐3‐Carboxamide (KT‐939)

For the synthesis of KT‐939, 3‐methyloxetane‐3‐carboxylic acid (46 mg, 0.40 mmol), N‐(3‐dimethylaminopropyl)‐N′‐ethylcarbodiimide hydrochloride (EDCI, 100 mg, 0.52 mmol), 1‐hydroxybenzotriazole (HOBT, 70 mg, 0.52 mmol), and triethylamine (TEA, 105 mg, 1.04 mmol) were dissolved in dichloromethane (DCM, 5 mL) and stirred at room temperature for 5 min. Next, 4‐(2‐aminothiazol‐4‐yl)‐1,3‐phenylene di(isopropyl carbonate) (100 mg, 0.26 mmol) was added. After stirring at room temperature for 5 h, the reaction was quenched with water (30 mL) and extracted with DCM (3 × 30 mL). The combined organic layers were washed with brine (30 mL), dried over anhydrous Na_2_SO_4_, filtered, and concentrated under reduced pressure. The residue was dissolved in a methanol/tetrahydrofuran mixture (1:1, 2 mL), followed by the addition of 4 N NaOH (0.5 mL). This mixture was stirred at room temperature for 1 h, then water (30 mL) was added. The solution was acidified to pH 4–5 with 2 N HCl and extracted with ethyl acetate (3 × 30 mL). The combined organic layers were washed with brine (30 mL), dried over anhydrous Na_2_SO_4_, filtered, and concentrated under reduced pressure. The residue was purified by preparative HPLC to yield KT‐939 (15 mg, 46.9% yield) as a white powder.

LCMS([M+H]^+^): 307.05. ^1^H NMR (400 MHz, DMSO‐*d*_*6*_) δ 12.26 (s, 1H), 10.81 (s, 1H), 9.50 (s, 1H), 7.68 (d, *J* = 8.4 Hz, 1H), 7.46 (s, 1H), 6.34–6.28 (m, 2H), 4.86 (d, *J* = 6.2 Hz, 2H), 4.37 (d, *J* = 6.2 Hz, 2H), 1.63 (s, 3H).

### 2.2 Cell Culture and Reagents

This study employed multiple human and murine cell lines for in vitro assays. The MNT‐1 human melanoma cell line (Cat. ZQ1100) was obtained from Zhongqiao Xinzhou Biotechnology Co. Ltd. (Shanghai, China) and cultured in high‐glucose DMEM medium (SH30022.01, HyClone) supplemented with 20% FBS (16000‐044, Gibco), 10% AMI‐V Medium (12055‐091, Gibco), 1% Non‐Essential Amino Acids (NEAA; 11140‐050, Gibco), and antibiotics (100 U/mL penicillin and 100 μg/mL streptomycin). Additional cell lines included the B16‐F10 murine melanoma cell line (Cat. CBP60337), HEK293T human embryonic kidney cells (SCSP‐502), the immortalized human keratinocyte line HaCaT (Cat. BNCC339817), and RAW264.7 murine macrophage‐like cells. These were sourced from Nanjing Kebai Biotechnology Co. Ltd. (Jiangsu, China), the Cell Bank of Chinese Academy of Sciences (Shanghai, China), BeNa Culture Collection (Beijing, China), and American Type Culture Collection (Manassas, USA), respectively. All cell lines were cultured in basal DMEM enriched with 10% FBS, 100 U/mL penicillin, and 100 μg/mL streptomycin at 37°C in a humidified atmosphere containing 5% CO_2_ and 95% air.

Thiamidol, glabridin, niacinamide, and arbutin were purchased from Bide Pharmatech Co. Ltd. (Shanghai, China); phenylethyl resorcinol, resveratrol, and kojic acid from Leyan Ltd. (Nanjing, China); 4‐butylresorcinol from Aikon Biopharmaceuticals (Jiangsu, China); hydroquinone and L‐DOPA from MedChem Express (Monmouth Junction, USA); pGL4.27 ARE NRF2‐SPE plasmid from Biofeng Bioscience (Beijing, China); Renilla luciferase plasmid from Thermo Fisher Scientific (Waltham, USA); lipopolysaccharide (LPS) from Sigma‐Aldrich (St. Louis, USA); and Lipofectamine 2000 from Invitrogen (Carlsbad, USA).

### 2.3 L‐DOPA Oxidation Assay for Tyrosinase Activity

The hTYR‐mediated L‐DOPA oxidation assay was performed according to Winder and Harris [17]. MNT‐1 cells were resuspended in 0.05 M sodium phosphate buffer (pH 6.9) and sonicated for 1 min. After incubation on ice for 3–5 min, the mixture underwent a second sonication. The lysate was centrifuged at 13 000 rpm for 15 min at 4°C, and the supernatant was collected and kept on ice.

For the assay, 20 μL of crude enzyme solution and 80 μL sodium phosphate buffer were added to a 96‐well plate and centrifuged at 1000 rpm for 30 s. Subsequently, 2 μL of different concentrations of test compounds were added to each well, followed by centrifugation at 1000 rpm for 30 s. Finally, 100 μL L‐DOPA (final concentration 4 mM) was added to initiate the reaction. The microplate reader (Synergy H1 Hybrid Reader, BioTek) was used to monitor the reaction at 26°C, measuring absorbance at 475 nm every minute for 60 min. IC_50_ values were calculated using GraphPad software based on Vmax values from the kinetic curves.

### 2.4 Evaluation of Melanin Content

MNT‐1 cells were exposed to varying concentrations of test compounds for 6 days. After treatment, cells were harvested and lysed in RIPA buffer on ice for 20 min. Lysates were centrifuged at 13 000 rpm for 10 min at 4°C, and the supernatant was collected. Protein concentration was quantified using a BCA Protein Assay Kit (Thermo Fisher). To extract melanin, cell pellets were dissolved in 1 M NaOH and incubated for 3 h at 80°C. Absorbance at 405 nm was measured using a Synergy H1 Hybrid Reader. Melanin content was quantified by referencing a standard curve of synthetic melanin (maximum concentration 50 μg/mL, 2‐fold gradient dilution, 8 concentration points). Melanin content was normalized to protein concentration (μg melanin/mg protein), and IC_50_ values were calculated using GraphPad software.

### 2.5 DPPH Radical Scavenging Assay

A DPPH radical scavenging assay kit (Solarbio) was used to assess antioxidant activity, following the manufacturer's instructions. Briefly, 10 μL of each test compound at the indicated concentrations was mixed with 190 μL DPPH solution in a 96‐well plate. Control wells (10 μL compound + 190 μL ethanol) and blank wells (10 μL solvent + 190 μL ethanol) were included. The reaction mixtures were gently shaken and incubated for 30 min at room temperature in the dark. Absorbance was measured at 515 nm using a Synergy H1 Hybrid Reader.

### 2.6 NRF2/ARE‐Luciferase Reporter Assay

The NRF2‐responsive firefly luciferase reporter plasmid (pGL4.27_ARE NRF2‐SPE) and Renilla luciferase control plasmid (pRL‐TK) were co‐transfected into HEK293T cells using Lipofectamine 2000. After 24 h, transfected cells were seeded into 96‐well plates and incubated overnight. Test compounds (10 μM) were added, and cells were incubated for an additional 48 h. Luciferase activities were measured using the Dual‐Lumi Luciferase Reporter Assay Kit (Beyotime Biotechnology), following the manufacturer's instructions.

### 2.7 RNA Extraction and RT‐qPCR Assay

RAW264.7 cells were treated with KT‐939 at concentrations of 3, 10, or 30 μM and incubated for 2 h. After pretreatment, cells were stimulated with 100 ng/mL LPS for an additional 16 h. Total RNA was extracted using the MiniBEST RNA Extraction Kit (Takara Bio) per the manufacturer's protocol. Reverse transcription and SYBR Green‐based qPCR were performed with ABScript II One Step RT‐qPCR Kit (Abclonal). RT‐PCR primer sequences were as follows: (Mouse) TNF‐α sense: 5‐GGTGCCTATGTCTCAGCCTCTT‐3, antisense: 5‐GCCATAGAACTGATGAGAGGGAG‐3; (Mouse) IL‐6 sense: 5‐TACCACTTCACAAGTCGGAGGC‐3, antisense: 5‐CTGCAAGTGCATCATCGTTGTTC‐3; (Mouse) IL‐1α sense: 5‐ACGGCTGAGTTTCAGTGAGACC‐3, antisense: 5‐CACTCTGGTAGGTGTAAGGTGC‐3; (Mouse) GAPDH sense: 5‐CATCACTGCCACCCAGAAGACTG‐3, antisense: 5‐ATGCCAGTGAGCTTCCCGTTCAG‐3; Expression of target gene mRNA was normalized to GAPDH, and results were analyzed using GraphPad software.

### 2.8 Cell Viability

B16‐F10, MNT‐1, and HaCaT cells (5000 cells/well) were seeded into 96‐well plates and incubated overnight. Culture medium (50 μL) containing varying concentrations of KT‐939 was added to each well, and cells were incubated for 72 h. Cell viability was assessed using the Cell Viability Assay Kit (Vazyme Biotech), following the manufacturer's instructions. Luminescence was measured using a Synergy H1 Hybrid Reader. DMSO‐treated cells served as the control, and survival rates were calculated and analyzed with GraphPad software.

### 2.9 Statistical Analysis

All data are presented as mean ± standard deviation (SD). A two‐tailed unpaired Student's *t*‐test was used for comparisons between two groups. One‐way ANOVA was used for analyses involving more than two groups. All statistical analyses were performed using GraphPad Prism 9. Statistical significance is indicated as *p < 0.01, **p < 0.05, and ***p < 0.001, unless otherwise specified.

### 2.10 Clinical Studies

A single‐center human study was conducted in 32 healthy Chinese female subjects with sensitive skin (aged 33–59 years, mean age 45.75 ± 6.90 years). All subjects completed the study. Each participant applied KOSHINE ANTI‐PIGMENT LOTION containing 0.2% KT‐939 to the entire face twice daily, morning and evening, after cleansing, for 28 consecutive days. A self‐control design was adopted. Facial assessments were performed before treatment and after 28 days. Laboratory technicians collected VISIA images, obtained skin parameter measurements using a Mexameter MX18, and administered questionnaires regarding product usage. Informed consent was obtained from all participants, and the study was approved by an independent ethics committee. KT‐939 activates the ARE/NRF2 signaling pathway. HEK293T cells transfected with an NRF2‐responsive firefly luciferase reporter were treated with 10 µM of KT‐939, hydroquinone (Hq), 377, or 4‐butylresorcinol for 48 h. Luminescence signals were normalized to renilla and expressed relative to DMSO control. *** *p* < 0.001 vs DMSO.

### 2.11 Molecular Modeling

Sequence information for hTYR was retrieved from UniProt (entry P14679). The structural model of hTYR was constructed using AlphaFold2 [18]. Three‐dimensional structures of Thiamidol and KT‐939 were built and optimized with the MMFF94 force field in RDKit. Molecular docking was performed using AutoDock Vina 1.2.1 [19], generating 10 binding poses for each molecule. Only the lowest energy conformation with plausible geometry was selected for further analysis.

## 3 Results

### 3.1 Inhibition of Human Tyrosinase (hTYR)

**FIGURE 1:**
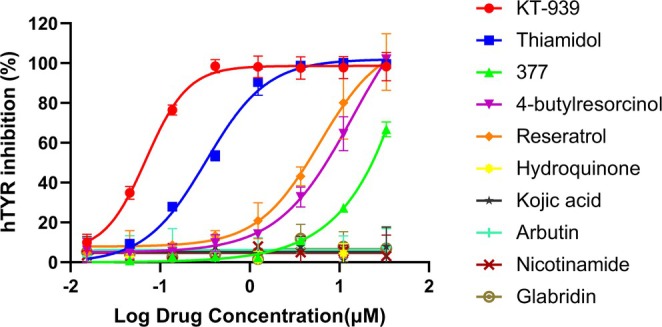
Inhibitory activity of KT‐939 against hTYR from MNT‐1 cell lysates. The L‐DOPA tyrosinase assay was performed using a tyrosinase enzyme solution derived from MNT‐1 cells in 50 mM sodium phosphate buffer (pH 6.9), with a substrate (L‐DOPA) concentration of 4 mM and various concentrations of inhibitors as indicated. Data represent mean ± standard deviation of three independent experiments.

Tyrosinase functions as a key catalytic enzyme and plays a crucial role in regulating melanin synthesis and pigmentation processes. To evaluate the inhibitory potential of KT‐939 on hTYR, we compared its activity with nine other tyrosinase inhibitors widely used in cosmetics and pharmaceuticals, including hydroquinone, arbutin, kojic acid, Thiamidol, phenethyl resorcinol (377), 4‐butylresorcinol, resveratrol, nicotinamide, and glabridin. Among these inhibitors, KT‐939 demonstrated superior inhibition of hTYR, with an IC_50_ of 0.07 μM and achieved nearly complete inhibition of hTYR at concentrations ≥ 0.4 μM. In comparison, the IC_50_ values of Thiamidol, 377, 4‐butylresorcinol, and resveratrol were 0.33, 21.28, 7.09, and 6.04 μM, respectively. At a concentration of 0.4 μM, the inhibitory activities of Thiamidol, 377, 4‐butylresorcinol, and resveratrol were only 53.5%, 2.7%, 9.7%, and 13.4%, respectively, compared to the control. Thus, KT‐939 exhibited approximately four‐fold greater potency than Thiamidol, 86‐fold greater than resveratrol, 100‐fold greater than 4‐butylresorcinol, and over 300‐fold greater than 377. Hydroquinone, kojic acid, arbutin, nicotinamide, and glabridin showed no significant inhibitory effects even at the maximum tested concentration of 33.3 μM (Figure 1). Collectively, KT‐939 is the most potent hTYR inhibitor reported to date.

### 3.2 Molecular Modeling and Structure–Activity Relationship (SAR Analysis)

**FIGURE 2:**
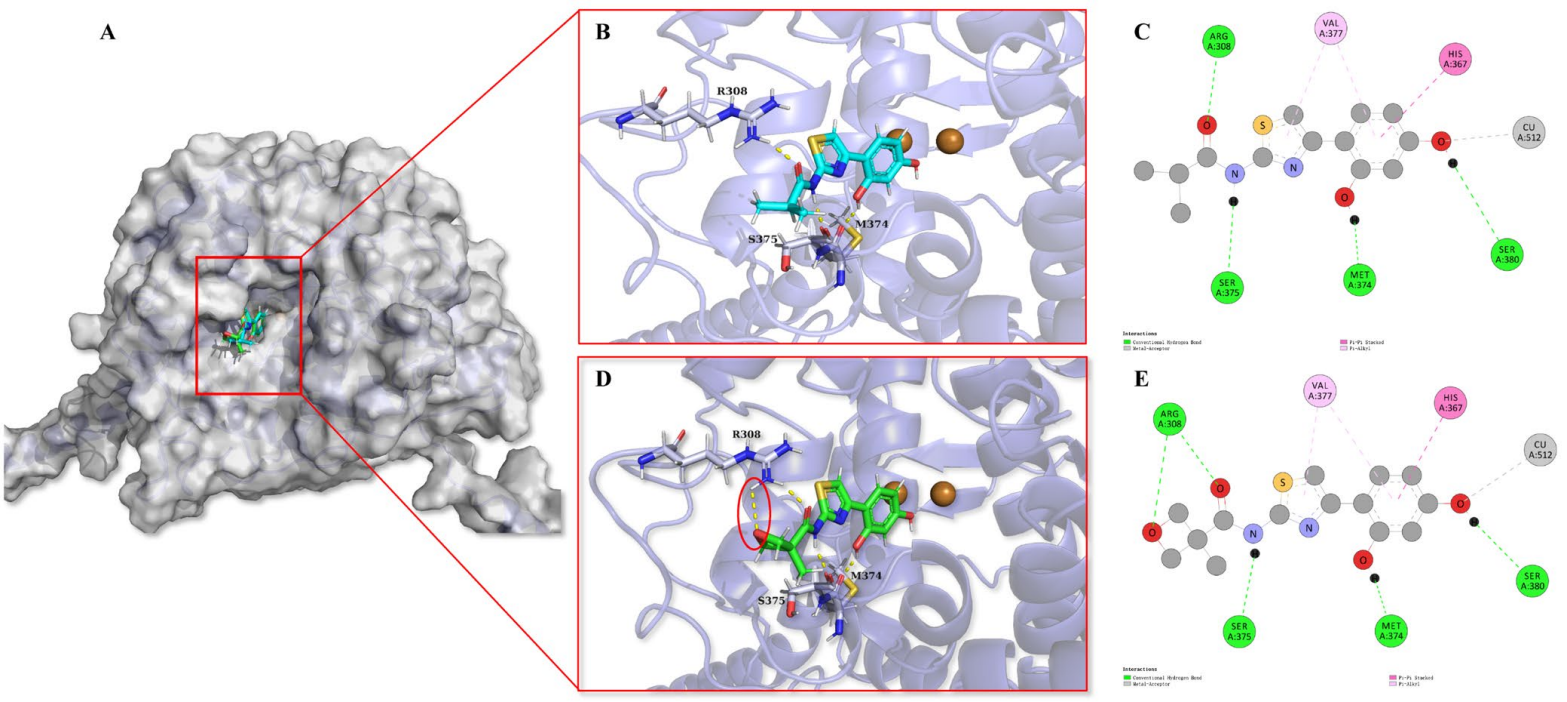
Molecular modeling and interaction patterns of Thiamidol. (A) Predicted pose obtained for Thiamidol (color cyan) and KT‐939 (color green). (B) Interaction pattern of Thiamidol‐hTYR. (C) Schematic view of key interactions predicted for the hTYR‐Thiamidol complex. (D) Interaction pattern of KT‐939‐hTYR. (E) Schematic view of key interactions predicted for the hTYR‐KT‐939 complex. Yellow dashed lines indicated hydrogen bonds. Red cycle region indicated an additional hydrogen bonding interaction compared to Thiamidol.

Molecular docking analysis was performed to investigate the binding modes of Thiamidol and KT‐939 to hTYR. As shown in Figure 2A, the best docking poses of both compounds displayed high similarity, with each establishing an extensive contact network within the hTYR catalytic pocket (Figure 2B–E). Consistent with previous studies, the 4‐hydroxyl group coordinates the catalytic Cu^2+^‐512 ion with precise geometric complementarity while forming a critical hydrogen bond with Ser380. Additionally, the 2‐hydroxyl group interacts with the backbone carbonyl of Met374, collectively stabilizing molecular anchoring within the active site.

Both inhibitors also reproduce the binding paradigm of native substrates (L‐tyrosine and L‐DOPA), engaging in face‐to‐face *π*–*π* stacking interactions with His367, a copper‐ligating residue, as observed in Zn^2+^‐soaked TYR crystal structures [20]. Hydrophobic stabilization is further provided by contacts between the resorcinol and thiazole moieties and the isopropyl side chain of Val377, contributing to strong shape complementarity within the catalytic cavity.

The amide linker plays a crucial role in scaffold stabilization, forming a bidentate anchoring system via an amide‐NH⋯Oγ hydrogen bond to Ser375 and a carbonyl‐O⋯Hη hydrogen bond to Arg308. These interactions are shared by both compounds; however, KT‐939 exhibits a distinctive structural advantage through its oxetane substituent, which forms an additional hydrogen bond with the guanidinium group of Arg308—an interaction absent in Thiamidol. This supplementary interaction likely accounts for the enhanced binding affinity and approximately four‐fold increase in potency observed with KT‐939.

Computational binding affinity calculations support these structural observations: KT‐939 achieves a superior docking score of −7.1 kcal/mol compared to Thiamidol's −6.9 kcal/mol, aligning with experimental IC_50_ values (KT‐939: 0.07 μM; Thiamidol: 0.33 μM). Collectively, these findings highlight the key pharmacophoric features—resorcinol hydroxyl groups, amide linker, thiazole ring, and oxetane substituent—that drive KT‐939's optimized inhibitory activity against human tyrosinase.

### 3.3 Inhibition of Melanin Production

To investigate the impact of tyrosinase inhibitors on melanin synthesis, the human melanoma cell line MNT‐1 was used to evaluate melanin production after six days of treatment. KT‐939 demonstrated potent inhibition of melanin production, with an IC_50_ of 0.36 μM. This represented approximately 3.6‐fold greater inhibitory potency compared to Thiamidol (IC_50_: 1.3 μM) and significantly outperformed other tyrosinase inhibitors such as 4‐butylresorcinol (IC_50_: 8.0 μM), 377, arbutin, hydroquinone, and kojic acid (data not shown).

**FIGURE 3:**
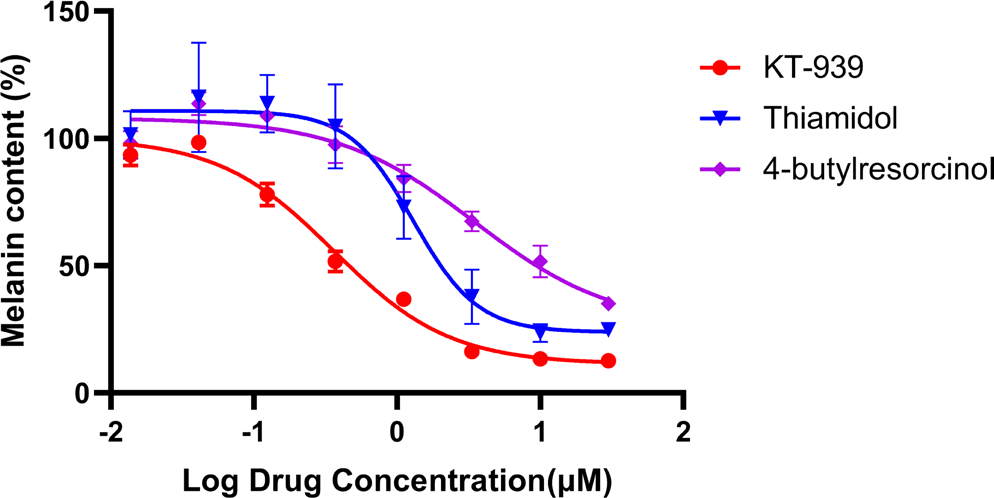
Inhibition of melanin production in MNT‐1 by KT‐939, Thiamidol, and 4‐butylresorcinol. MNT‐1 cells were treated with different concentrations of KT‐939, Thiamidol, or 4‐butylresorcinol for 6 days. The melanin content was quantified based on the standard curve of melanin, and the melanin content per mg of protein was utilized for IC_50_ calculations with GraphPad software.

At a concentration of 0.37 μM, neither Thiamidol nor 4‐butylresorcinol showed inhibitory activity, whereas KT‐939 reduced melanin production by 48.4%. At concentrations of 1.1 μM and 3.3 μM, KT‐939 inhibited melanin production by 63.3% and 83.9%, respectively, compared with 27.2% and 62.2% for Thiamidol, and 15.8% and 32.6% for 4‐butylresorcinol. At a concentration of 10 μM, KT‐939 inhibited melanin production by 86.7%, while Thiamidol and 4‐butylresorcinol inhibited melanin production by 76.5% and 48.4%, respectively (Figure 3). As a novel and highly potent tyrosinase inhibitor, KT‐939 exhibits strong melanin inhibitory activity at very low concentrations, far surpassing other skin‐lightening agents.

**FIGURE 4:**
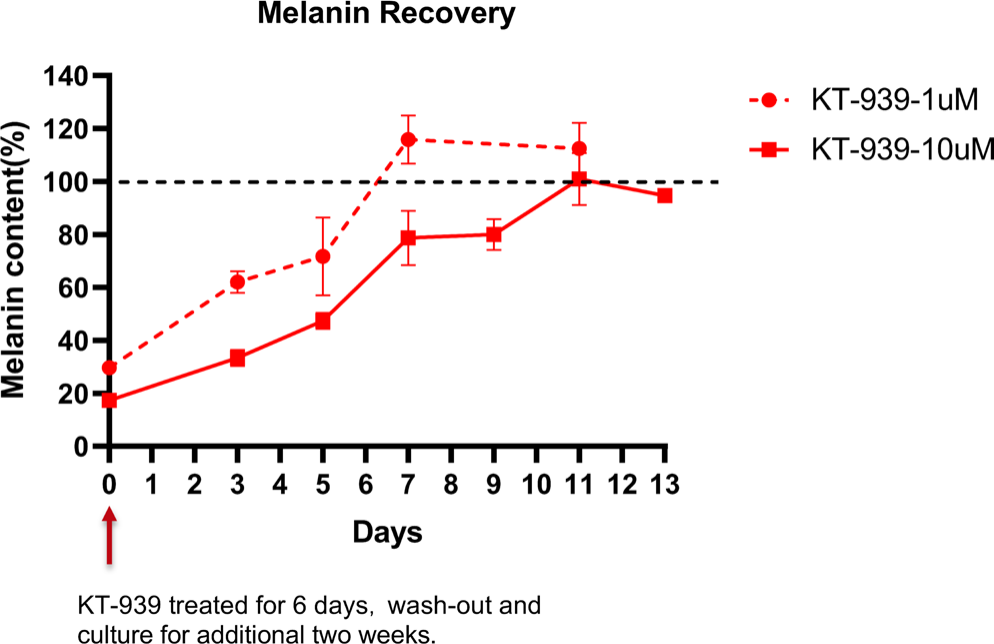
The inhibitory effect of KT‐939 on melanin production is reversible. MNT‐1 cells were exposed to 1 or 10 μM of KT‐939 for 6 days, followed by drug washout and replacement with normal medium. Melanin content was measured at specified time points. Data represent the mean ± standard deviation of three independent experiments.

To assess whether KT‐939 possesses long‐term inhibitory activity, a wash‐out assay was performed, and melanin production was measured over time. MNT‐1 cells were treated with 1 or 10 μM KT‐939 for six days, after which the drug‐containing medium was replaced with standard culture medium. Melanin content was measured at various time points. After the washout of 1 μM KT‐939, the melanin inhibition rate was 38% on day 3 and 28.3% on day 5, returning to baseline after seven days. Following the washout of 10 μM KT‐939, melanin inhibition rates were 66.6% and 52.7% on days 3 and 5, and 21.3% and 20% on days 7 and 9, respectively. Melanin content fully returned to normal levels after 11 days of drug withdrawal (Figure 4). These results indicate that the inhibitory effect of KT‐939 on melanin production is reversible upon the removal of the compound.

### 3.4 Antioxidant Activity of KT‐939

**FIGURE 5:**
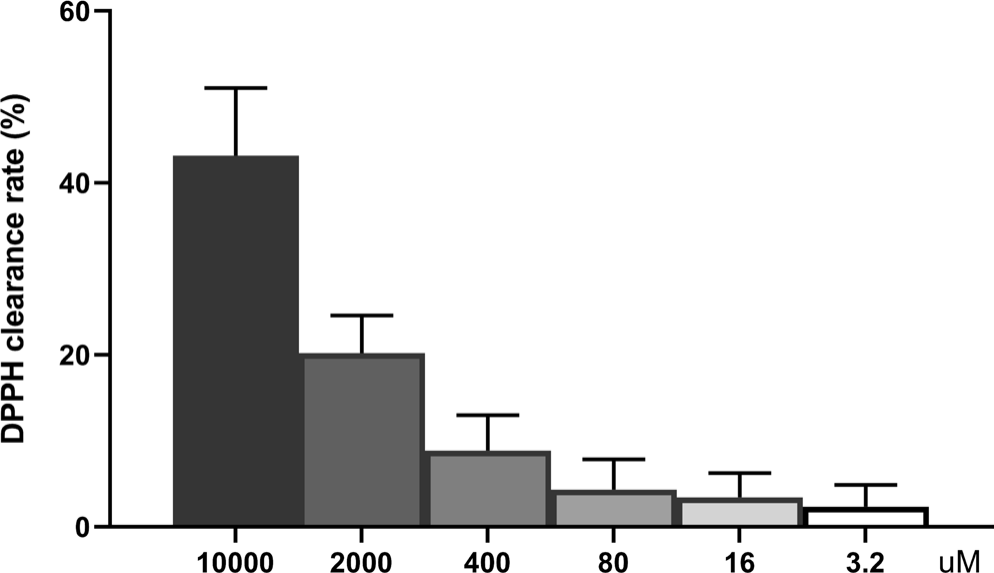
KT‐939 demonstrates significant free radical scavenging capacity. A DPPH radical scavenging assay was conducted to evaluate the antioxidant activity of KT‐939 at various concentrations, with a maximum concentration of 10 mM in a 5‐fold dilution series. Data represent mean ± standard deviation of three independent experiments.

During normal metabolism, the body generates free radicals, and its ability to scavenge these declines with age. Additional factors such as ultraviolet radiation, irregular lifestyles, smoking, alcohol consumption, mental stress, emotional issues, air pollution, and radiation further increase free radical production [21, 22]. Excessive free radicals accelerate collagen cross‐linking and polymerization, promote elastic fiber degradation, reduce skin firmness and elasticity, and lead to wrinkles, dry keratosis, and rough, dull skin [23]. To explore the potential application of KT‐939 in skin management, a DPPH clearance assay was used to investigate free radical scavenging activity. KT‐939 displayed dose‐dependent DPPH radical scavenging capacity, with clearance rates of 43.2%, 20.2%, and 8.9% at concentrations of 10, 2, and 0.4 mM, respectively (Figure 5).

**FIGURE 6:**
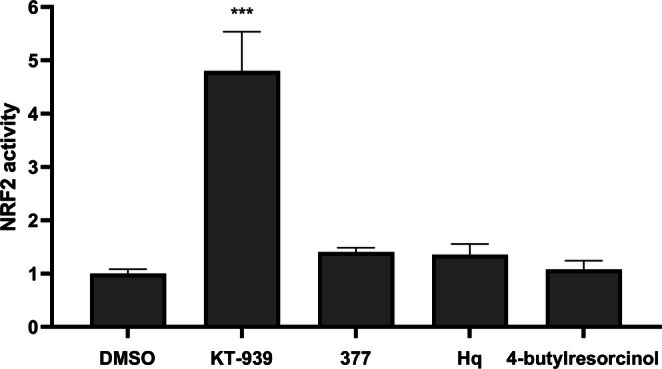
KT‐939 activates the ARE/NRF2 signaling pathway. HEK293T cells transfected with an NRF2‐responsive firefly luciferase reporter were treated with 10 µM of KT‐939, hydroquinone (Hq), 377, or 4‐butylresorcinol for 48 h. Luminescence signals were normalized to renilla and expressed relative to DMSO control. *** *p* < 0.001 vs DMSO.

The nuclear factor E2‐related factor 2 (NRF2)/ARE signaling pathway functions as a central defense mechanism against oxidative stress, inflammation, and toxic damage [24]. It maintains intracellular REDOX homeostasis by regulating antioxidant protein expression and detoxification enzymes. Given NRF2's pivotal role in antioxidant defense, the NRF2/ARE luciferase reporter system was used to determine whether KT‐939 activates NRF2 signaling. The results demonstrated that KT‐939 significantly enhanced the NRF2 signaling pathway, whereas other tyrosinase inhibitors, including hydroquinone, 377, and 4‐butylresorcinol, had no effect on NRF2 activation (Figure 6). These findings suggest that KT‐939 not only directly scavenges free radicals but also exerts antioxidant effects through activation of the NRF2 signaling pathway.

### 3.5 Anti‐Inflammatory Effects of KT‐939

**FIGURE 7:**
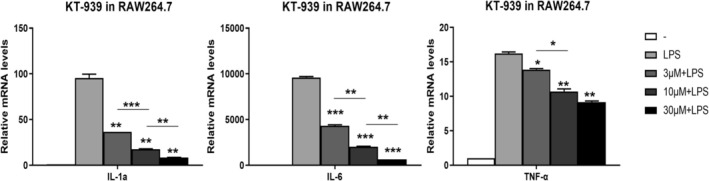
KT‐939 exhibits anti‐inflammatory effects. RAW264.7 cells were pre‐treated with different concentrations of KT‐939, followed by the addition of 100 ng/mL LPS for 16 h. mRNA levels of TNF‐α, IL‐6, and IL‐1α were quantified by RT‐PCR and compared to the LPS‐induced group (*p < 0.05, **p < 0.01, ***p < 0.001).

In addition to its antioxidant properties, we investigated whether KT‐939 exhibits anti‐inflammatory effects. To evaluate the regulatory role of KT‐939 on immune responses, an LPS‐induced inflammation injury model was established in RAW264.7 cells. Cells were treated with varying concentrations of KT‐939, followed by overnight incubation with LPS‐containing medium. Total RNA was extracted, and RT‐PCR was performed to examine the expression of several inflammatory‐related cytokines, including interleukin (IL)‐6, IL‐1α, and tumor necrosis factor (TNF)‐α. Compared with the LPS‐stimulated control group, KT‐939 treatment resulted in a dose‐dependent decrease of IL‐6, IL‐1α, and TNF‐α mRNA levels (Figure 7). These findings suggest that KT‐939's anti‐inflammatory activity may be attributed to its ability to downregulate expression of key pro‐inflammatory mediators.

### 3.6 In Vitro Safety Evaluation of KT‐939

**FIGURE 8:**
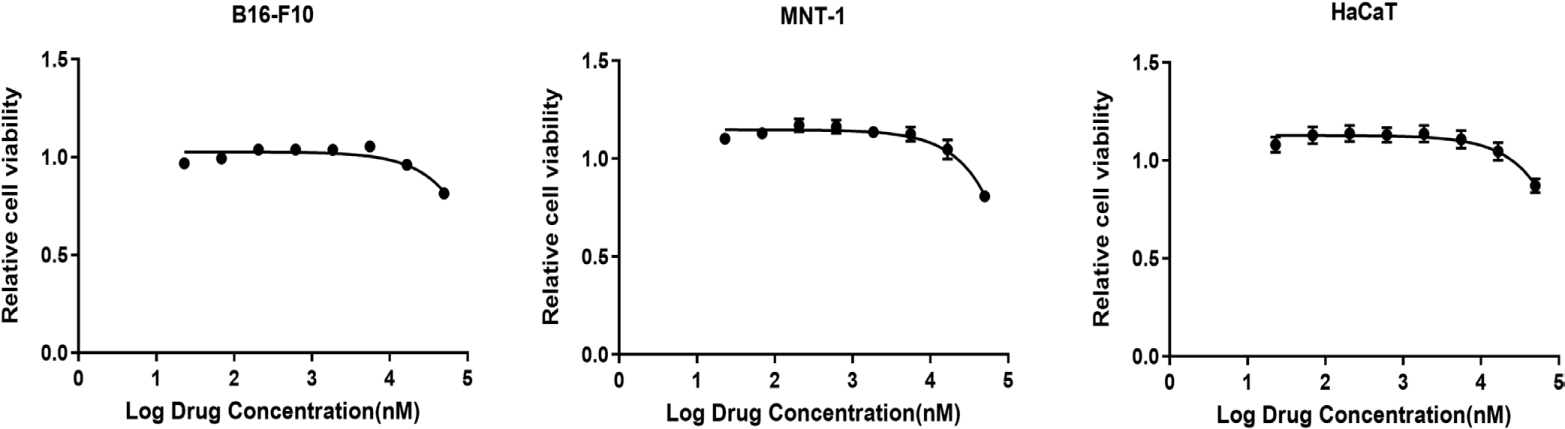
KT‐939 shows no cytotoxicity on B16‐F10, MNT‐1, and HaCaT cells. B16‐F10, MNT‐1, and HaCaT cells were incubated with different concentrations of KT‐939 for 72 h. Cell viability was assessed and normalized to the DMSO‐treated control group.

As a potent tyrosinase inhibitor, KT‐939 not only suppresses melanin production but also demonstrates antioxidant and anti‐inflammatory properties. The in vitro safety profile of KT‐939 was evaluated in skin‐related cell lines, including human melanoma MNT‐1, murine melanoma B16‐F10, and immortalized human keratinocyte HaCaT. These cells were exposed to various concentrations of KT‐939 for 72 h, and cell viability was measured. KT‐939 exhibited no significant cytotoxicity in B16‐F10, HaCaT, and MNT‐1 cells, with cell viability remaining unaffected even at concentrations up to 50 μM. This finding suggests that KT‐939 is well‐tolerated in vitro under the tested conditions (Figure 8).

### 3.7 Clinical Studies

**FIGURE 9:**
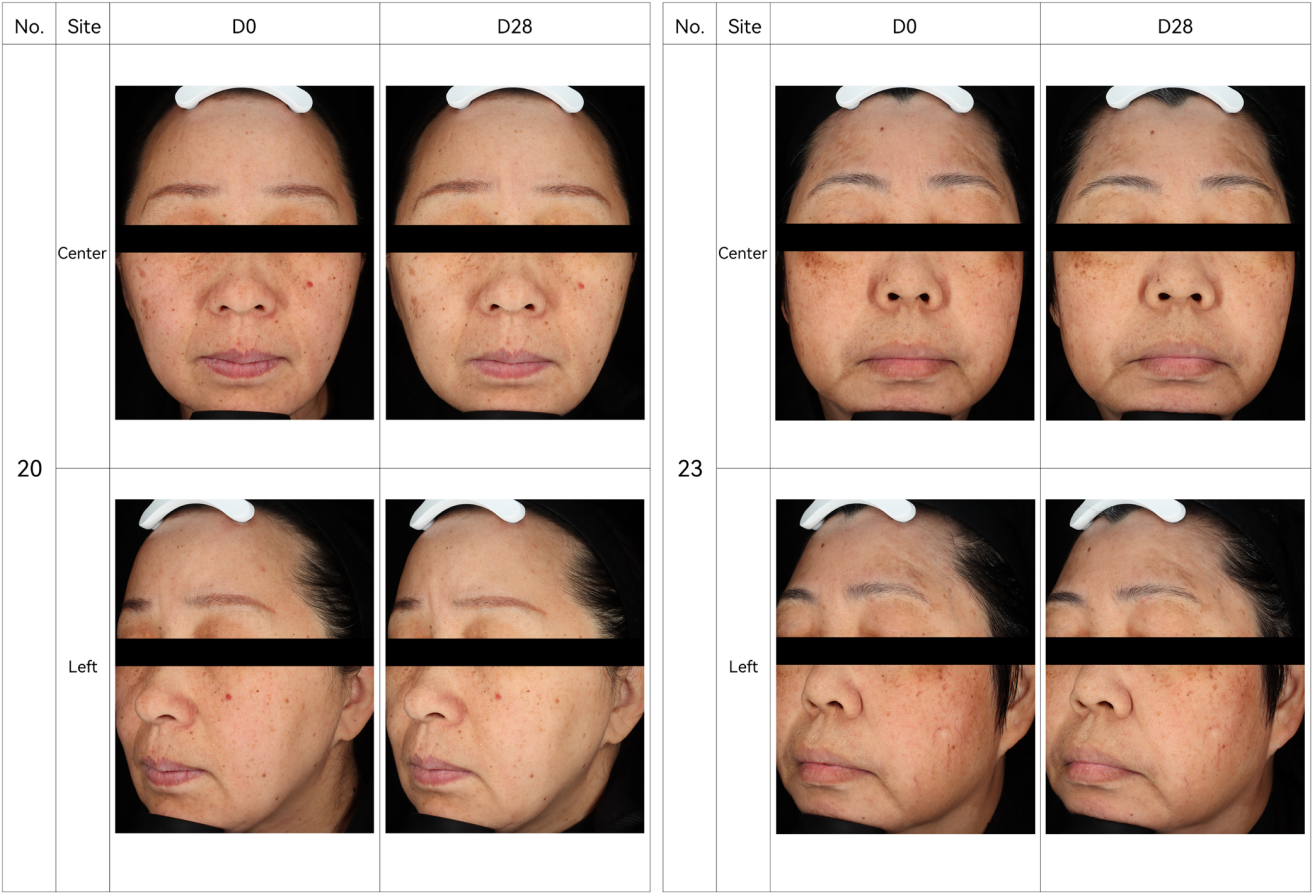
The lotion containing 0.2% KT‐939 has the effects of fading skin spots, increasing skin tone evenness, and brightening skin tone. Facial images were acquired and analyzed using VISIA 7 in combination with Image Pro‐Plus comprehensive skin analysis software. Representative comparison images under standard light from VISIA 7 after 28 days of product use are shown below (Subject No. 20 and 23).

A clinical study was conducted to evaluate KT‐939 efficacy in vivo. Participants applied a topical formulation containing 0.2% KT‐939 to the entire face twice daily for 28 consecutive days. After 28 days of treatment, significant improvements were observed in skin evenness, gloss, skin color, and melanin content compared to baseline (Day 0) (Table 1). Subjects' self‐assessment also indicated marked improvements in skin whiteness, reduction of skin spots, and decreased skin sensitivity, with statistically significant differences compared to baseline (*p* < 0.001) (Table 2). Satisfaction assessment showed that 94%–100% of participants reported satisfaction with the product (Table 3). Overall, the product demonstrated effects of fading skin spots, increasing skin tone evenness, and enhancing brightness, while remaining mild, non‐irritating, and suitable for sensitive skin (Figure 9). No adverse events (AEs) were observed during the study.

**TABLE 1:** Equipment‐based measurement.

Parameter (equipment) (site)	Time point	Rate of change	Significant *p*‐value compared with D0	Significant difference compared with D0	Subjects Improved *n* (improved %)	Description of parameters
Skin tone evenness (VISIA 7&IPP) (Pigmented side cheek)	D28	−2.66%	0.005	Significantly	27 (84%)	A lower value indicates improved evenness
Skin gloss (VISIA 7&IPP) (Cheek)	D28	16.55%	< 0.001	Significantly	25 (78%)	A higher value indicates improved gloss
Skin tone L* value (VISIA 7&IPP) (Cheek)	D28	1.01%	0.001	Significantly	25 (78%)	A higher value indicates brighter skin
Skin tone ITA° value (VISIA 7&IPP) (Cheek)	D28	2.61%	< 0.001	Significantly	25 (78%)	A higher value indicates lighter skin
Skin melanin content (Mexameter MX18) (Pigmentation area)	D28	−4.60%	< 0.001	Significantly	31 (97%)	A lower value indicates reduced melanin

**TABLE 2:** Self‐assessment (skin condition).

Item	Time point	Change value	Significant *p*‐value versus D0	Significance versus D0	Description of parameters
Whiteness of facial skin	D28	−3.03	< 0.001	Significantly	Evaluation standard: score 0 to 9, 0 score = very good, 1–3 scores = relatively good, 4–5 scores = good, 6–8 scores = average, 9 scores = very poor.
Degree of facial pigmentation	D28	−3.22	< 0.001	Significantly	
Glossiness of facial skin	D28	−3.03	< 0.001	Significantly	
Frequency of facial skin itching	D28	−2.97	< 0.001	Significantly	
Sensitivity of facial skin	D28	−3.03	< 0.001	Significantly	
Facial skin irritation/discomfort	D28	−3.16	< 0.001	Significantly	

**TABLE 3:** Self‐assessment (satisfaction).

Questions	Satisfaction
	D28 (%)
The sample can brighten and whiten the skin	97
The sample can brighten skin tone and improve dullness of the skin	94
The sample helps to lighten discoloration	97
The sample can reduce pigmentation	97
The sample is mild and non‐irritating	100
The sample is suitable for sensitive skin	100
The sample has a soothing effect	97
The sample can smooth skin	94
The sample provides a tightening and refining effect	94

*Note:* Standards of grading: 1–5 score, 5 = satisfied, 4 = relatively satisfied, 3 = commonly, 2 = relatively dissatisfied, and 1 = dissatisfied. Satisfaction = (number of subjects with score > 3 score + number of valid subjects) × 100%.

## 4 Discussion

Skin pigmentation disorders represent a major dermatological concern worldwide. The treatment of hyperpigmentation—including age spots, freckles, melasma, sunspots, and inflammation‐related hyperpigmentation—is often challenging, time‐consuming, and requires considerable patience as well as an in‐depth understanding of various therapeutic modalities to achieve success [25]. Among these conditions, melasma, characterized by excessive melanin accumulation, is a chronic disorder that typically occurs on sun‐exposed areas of the skin and is strongly associated with ultraviolet (UV) radiation or other contributing factors [26]. Currently, topical medications are commonly used to manage melasma, including agents such as tretinoin, hydroquinone, glucocorticoids, and other skin‐lightening compounds [27]. While the combination of hydroquinone with retinoids remains one of the most effective regimens for melasma, clinical outcomes are often limited by frequent recurrences or adverse reactions, including erythema, skin irritation, and inflammation‐induced hyperpigmentation [28]. These limitations underscore the ongoing need for safer and more effective therapeutic approaches for melasma and other hyperpigmentation‐related conditions. Within the melanogenesis pathway, the catalysis of DOPA by tyrosinase represents the critical rate‐limiting step. Therefore, novel strategies targeting tyrosinase are urgently required to address current clinical challenges. Safe and effective hTYR inhibitors are anticipated to either replace hydroquinone as the next generation of therapeutic agents or be widely utilized in the cosmetic field.

Currently, screening for tyrosinase inhibitors typically relies on cell‐based assays or mTYR assays in vitro. However, these approaches are limited by high cost, time‐intensiveness, or lack of specificity for hTYR. Obtaining hTYR protein remains challenging, which restricts its application and evaluation in tyrosinase inhibitor research. Tobias Mann and colleagues successfully expressed and purified hTYR and subsequently evaluated the activity of previously identified tyrosinase inhibitors against both mushroom and human tyrosinase. They discovered that inhibitors identified through mTYR‐based screening showed weak or negligible inhibitory activity against hTYR. Through high‐throughput screening of 50 000 compounds, they identified Thiamidol, which exhibited an IC_50_ of 1.1 μM against the hTYR enzyme and 0.9 μM in the MelanoDerm skin model [10]. In a clinical evaluation of patients with moderate‐to‐severe melasma and Fitzpatrick skin phototypes III–V, Thiamidol demonstrated favorable tolerability and significantly reduced melasma severity compared to both baseline and vehicle after 24 weeks of application [29]. Importantly, no significant difference in melasma improvement was observed between 0.2% Thiamidol cream and 4% hydroquinone cream [30], suggesting Thiamidol as a viable alternative for patients intolerant to or unresponsive to hydroquinone treatment.

In this study, we developed a simplified, cell lysate‐based assay for hTYR, enabling the entire process from lysate preparation using the MNT‐1 melanoma cell line to inhibitor screening to be completed in less than two hours. This approach has also been validated by other researchers [31]. Using this innovative method, we identified KT‐939 as the most effective tyrosinase inhibitor from a series of compounds. KT‐939 demonstrated an IC_50_ value of 0.07 μM for hTYR, significantly surpassing the efficacy of Thiamidol, 4‐butylresorcinol, 377, and other widely used tyrosinase inhibitors in cosmetics and pharmaceuticals, such as hydroquinone, arbutin, kojic acid, and resveratrol. Furthermore, KT‐939 exhibited robust inhibition of melanin production with an IC_50_ of 0.36 μM, substantially more potent than Thiamidol and 4‐butylresorcinol.

Importantly, the suppression of melanin production by KT‐939 was reversible, with normal levels restored within one to two weeks after drug withdrawal, thereby avoiding permanent depigmentation and enhancing its safety profile for cosmetic applications. Additionally, KT‐939 exhibited a DPPH clearance rate exceeding 40% and activated the NRF2 signaling pathway, exerting antioxidant activity through multiple mechanisms. KT‐939 also downregulated the expression of pro‐inflammatory cytokines such as IL‐6, IL‐1α, and TNF‐α. In a clinical study, the application of a lotion containing 0.2% KT‐939 for 28 days resulted in significant improvements in skin spot lightening, skin tone evenness, and overall brightness. Additionally, the formulation was well tolerated, mild, and non‐irritating, making it suitable for sensitive skin. As a potential compound targeting hyperpigmentation, KT‐939 demonstrates potent tyrosinase inhibition along with anti‐inflammatory and antioxidant properties, supporting its potential application in the treatment of melasma and other hyperpigmentation disorders, as well as its development as a whitening agent for cosmetic use.

## Author Contributions

X.H., Y.L., Q.Z., H.Y., X.Z., Y.Y., X.C., J.W., M.D., D.C., J.C., and Y.T. conducted the research, analyzed the data, and contributed to writing the paper. A.G. supervised the research, analyzed the data, and contributed to writing the paper. N.M. contributed to writing the paper. All authors have read and approved the final manuscript.

## Ethics Statement

The clinical study was reviewed and approved by SGS‐CSTC Standards Technical Services Co. Ltd.

## Consent

Informed consent was obtained from all participants, and the study was approved by SGS‐CSTC Standards Technical Services Co. Ltd.

## Conflicts of Interest

The authors declare no conflicts of interest.

## Data Availability

The datasets generated and/or analyzed during the current study are available from the corresponding author on reasonable request.
